# Supplementary material for: Genomic analysis of Plasmodium vivax field isolates circulating in sub-Saharan Africa
Source: Commun Biol. 2025 Jul 7;8:1012. doi: 10.1038/s42003-025-08276-5 (PMC12234883; doi:10.1038/s42003-025-08276-5)
Supplement: Supplementary file 2 — Supplementary Information [file 42003_2025_8276_MOESM2_ESM.pdf]

Genomic analysis of *Plasmodium vivax* field isolates circulating in sub-Saharan Africa

Supplementary information

Isabelle Bouyssou[1,2,3*] Lemu Golassa[4], Inès Vigan-Womas[5], Matthieu Schoenhals[6], Arsène Ratsimbasoa[7], Ali Ould Mohamed Salem Boukhary[8], Maria de Fátima Ferreira-da-Cruz[9], Sandrine Houzé[10], Laurence Ma[11], Feng Lu[12], Chetan Chitnis[3], Pascal Campagne[13*], Didier Ménard[1,3,14,15,16*]

[1] Institut Pasteur, Université Paris Cité, Malaria Genetic and Resistance Unit, INSERM U1201, F-75015 Paris, France

[2] Sorbonne Université, École Doctorale ED515 « Complexité du vivant », F-75005 Paris, France

[3] Institut Pasteur, Université Paris Cité, Malaria Parasite Biology and Vaccines Unit, F-75015 Paris, France.

[4] Aklilu Lemma Institute of Pathobiology, Addis Ababa University, Addis Ababa, Ethiopia

[5] Institut Pasteur de Dakar, Dakar, Senegal.

[6] Immunology of Infectious Diseases, Institut Pasteur Madagascar, Antananarivo, Madagascar

[7] Faculté de Médecine, Université de Fianarantsoa, Fianarantsoa, Madagascar

[8] Université de Nouakchott, Unité de recherche génomes et milieux, Nouakchott, Mauritania

[9] Fundacão Oswaldo Cruz, Malaria Research Laboratory, Rio de Janeiro, Brazil

[10] Centre National de Référence du Paludisme, Hôpital Bichat, F-75018 Paris, France

[11] Institut Pasteur, Université Paris Cite, Biomics Platform, C2RT, F-75015 Paris, France

[12] Department of Pathogenic Biology and Immunology, School of Medicine, Key laboratory of Jiangsu province university for Nucleic Acid & Cell Fate Manipulation, Affiliated Hospital of Yangzhou University, Yangzhou University, Yangzhou, China,

[13] Institut Pasteur, Université Paris Cité, Hub de Bio-informatiques et Biostatistique, Département Biologie Computationnelle, USR 3756, F-75015, Paris, France

[14] Université de Strasbourg, UR3073 – PHAVI - Pathogens Host Arthropods Vectors Interactions Unit, F-67000 Strasbourg, France
[15] CHU Strasbourg, Laboratory of Parasitology and Medical Mycology, F-67000 Strasbourg, France
[16] Institut universitaire de France (IUF), F-75231, Paris, France

Corresponding authors *
Didier Menard (dmenard@pasteur.fr or dmenard@unistra.fr), Isabelle Bouyssou (isabelle.bouyssou@pasteur.fr), Pascal Campagne (pascal.campagne@pasteur.fr).

Table of contents

1. Supplementary methods 4

2. Supplementary Figures 6

2.1. Figure S1. Depth coverage analysis........ 6

2.2. Figure S2. Within-Host Diversity Assessment Using the FWS Test........ 7

2.3. Figure S3. Global genetic diversity of P. vivax populations (monoclonal and polyclonal infections). 8

3. Supplementary Tables 9

3.1. Table S1: List of *P. vivax* genomic sequences already published and used in this study 9

3.2. Table S2: Distribution of monoclonal (Fws > 0.95) and polyclonal (Fws ≤ 0.95) isolates, by country of origin. 13

3.3. Table S3. Mutation points detected in genes associated with drug resistance in *P. vivax* isolates collected in the Comoros, Madagascar, Mauritania, Ethiopia and Djibouti........ 14

3.4. Table S4. Mutation points detected in invasion-related genes in *P. vivax* isolates collected in the Comoros, Madagascar, Mauritania, Ethiopia, and Djibouti. 15

3.5. Table S5. List of validated and suspected *P. vivax* invasion-related genes used in this study. 16

3.6. Table S6. Primer sequences and PCR conditions used in the study. 17

4. References 19

1. Supplementary methods

1.1. Variant Calling – Technical details (related to Methods > Variant Calling)

Reads were aligned to the *P. vivax* PvP01 reference genome (v48) using BWA-MEM with default parameters. Reads with MAPQ <30, secondary alignments, and insert sizes >1000 bp were excluded using SAMtools and in-house scripts. Duplicates were marked with Picard MarkDuplicates (v2.18.27). SNPs were called per sample using GATK HaplotypeCaller (v4.1.7.0) in diploid mode, followed by joint genotyping with GenotypeGVCFs. Variants were filtered using VariantFiltration with the following options: --filter-expression "QD < 2.0 || MQ < 40.0 || FS > 60.0 || SOR > 3.0 || MQRankSum < -12.5 || ReadPosRankSum < -8.0". Only biallelic SNPs were retained using --max-alleles 2, and genotypes with DP ≤4 were masked as missing. Variants with >10% missingness were excluded using VCFtools (v0.1.16). Final VCFs were merged with Picard GatherVcfs.

1.2. Population structure analysis (related to Methods > Population structure and genetic clustering)

Pairwise genetic distances were computed from the filtered biallelic SNP matrix using custom R scripts. Principal coordinate analysis (PCoA) was conducted with the ade4::dudi.pco() function in R (v4.3.0). To account for unequal sample sizes, a between-group analysis was performed to minimize group imbalance. Clustering was inferred with fastSTRUCTURE (v1.0), using the --prior simple setting, across a range of K values (K = 2 to 10). The optimal number of clusters was determined with the chooseK.py utility. Only monoinfections were included in these analyses.

1.3. Genomic differentiation and selection (related to Methods > Genomic differentiation and selection)

Nei's GST was calculated per SNP using the hierfstat package in R. Local smoothing of GST values was performed using a 100-SNP rolling mean to visualize genomic islands of differentiation. The statistical significance of peaks was assessed via a permutation approach: within each iteration, GST values were shuffled within chromosomes, smoothed, and the maximum genome-wide value recorded. Repeating this procedure generated a null distribution to assess peak significance. Gene-level GST was computed for coding regions of known invasion-related genes. Tajima's D was calculated for each gene using PopGenome, allowing comparison between invasion-related and genome-wide diversity patterns.

1.4. Genotype–phenotype association (related to Methods > Genotype–phenotype association analysis)

GLM analyses were conducted using the glm() function in R (binomial family, logit link). The model tested associations between parasite genotypes and host Duffy status while adjusting for country of origin: glm(Duffy_status ~ genotype + country, family = binomial).

Only monoinfections with known Duffy genotypes and SNPs with MAF >0.05 and <10% missingness were analyzed. Bonferroni correction was applied to adjust p-values for multiple testing using the p.adjust() function in R.

2. Supplementary Figures

2.1. Figure S1. Depth coverage analysis.

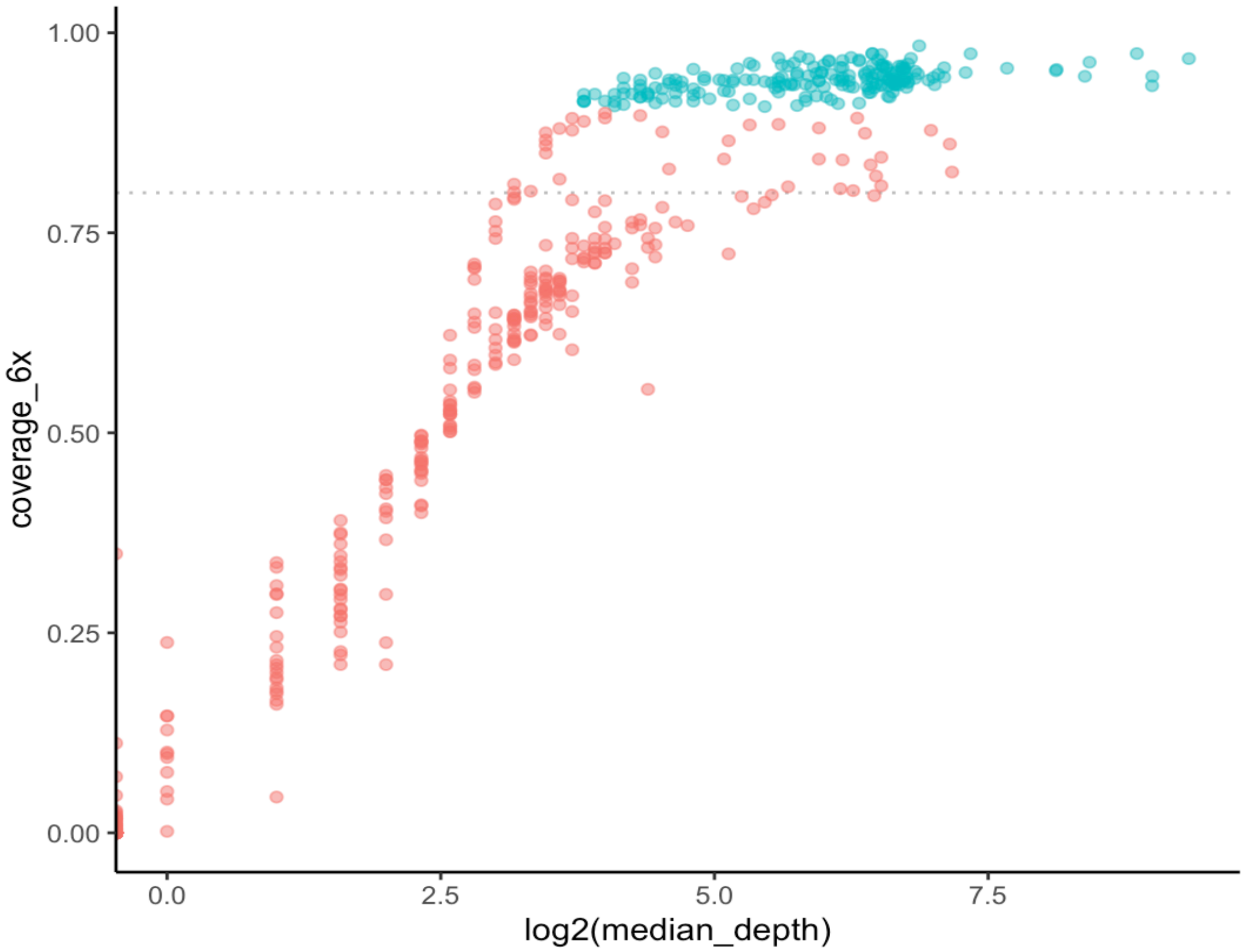

The number of samples above 80% (x6) was 276/751.

2.2. Figure S2. Within-Host Diversity Assessment Using the FWS Test.

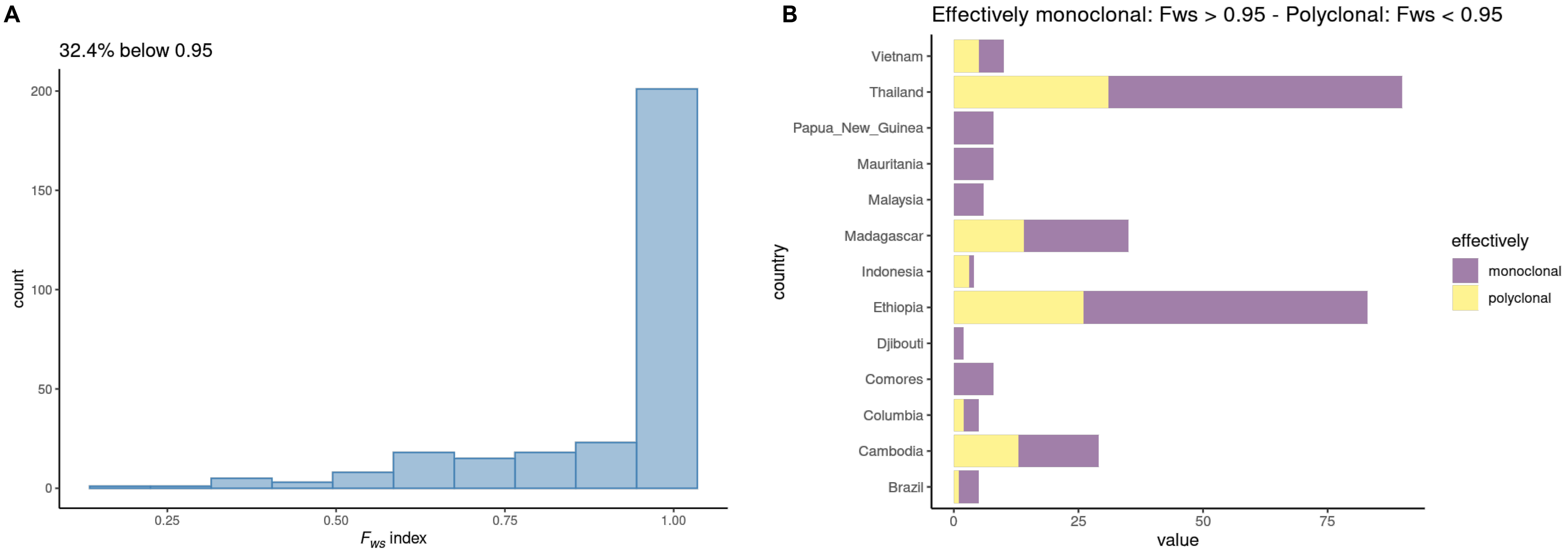

A. Distribution of F*ws* values across the analyzed samples. Each point represents an individual sample, with F*ws* values plotted along the x-axis. Samples with an F*ws* value below 0.95 are classified as polyclonal, while those above this threshold are considered monoclonal. **B.** Histogram of within-host diversity (F*ws* values). The x-axis shows F*ws* values, while the y-axis represents the number of samples within each value range.

2.3. Figure S3. Global genetic diversity of P. vivax populations (monoclonal and polyclonal infections).

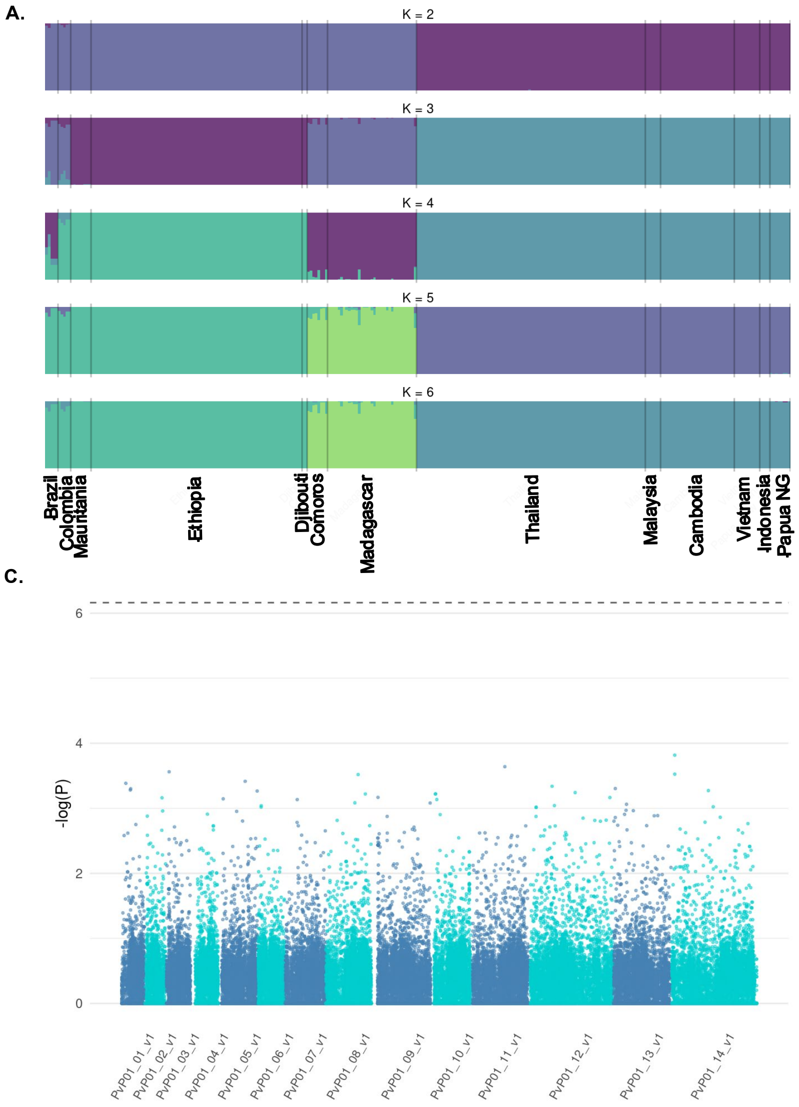

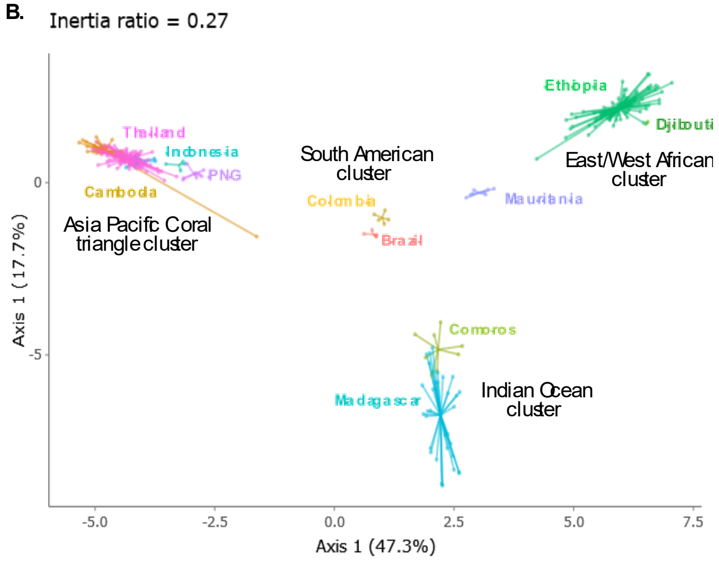

A. Structural analysis of chromosomes. **B.** Principal Coordinate analysis (PCO). **C.** Manhattan plot displaying the genome-wide association results.

3. Supplementary Tables

3.1. Table S1: List of *P. vivax* genomic sequences already published and used in this study

Country	ENA accession number	Reference	Ref
Brazil	ERR018932	Hupalo et al., Nature Genetics 2016	26
Brazil	ERR019040	Hupalo et al., Nature Genetics 2016	26
Brazil	SRR332567	Hupalo et al., Nature Genetics 2016	26
Brazil	SRR332569	Hupalo et al., Nature Genetics 2016	26
Brazil	SRR340133	Hupalo et al., Nature Genetics 2016	26
Columbia	SRR1562975	Hupalo et al., Nature Genetics 2016	26
Columbia	SRR1564650	Hupalo et al., Nature Genetics 2016	26
Columbia	SRR1564664	Hupalo et al., Nature Genetics 2016	26
Columbia	SRR1564665	Hupalo et al., Nature Genetics 2016	26
Columbia	SRR1567977	Hupalo et al., Nature Genetics 2016	26
Cambodia	ERR020103	MalariaGEN P. vivax Genome Variation Project, Wellcome Open Res 2022	23
Cambodia	ERR023039	MalariaGEN P. vivax Genome Variation Project, Wellcome Open Res 2022	23
Cambodia	ERR023040	MalariaGEN P. vivax Genome Variation Project, Wellcome Open Res 2022	23
Cambodia	ERR023041	MalariaGEN P. vivax Genome Variation Project, Wellcome Open Res 2022	23
Cambodia	ERR023042	MalariaGEN P. vivax Genome Variation Project, Wellcome Open Res 2022	23
Cambodia	ERR027119	MalariaGEN P. vivax Genome Variation Project, Wellcome Open Res 2022	23
Cambodia	ERR039234	MalariaGEN P. vivax Genome Variation Project, Wellcome Open Res 2022	23
Cambodia	ERR054080	MalariaGEN P. vivax Genome Variation Project, Wellcome Open Res 2022	23
Cambodia	ERR054082	MalariaGEN P. vivax Genome Variation Project, Wellcome Open Res 2022	23
Cambodia	ERR111729	MalariaGEN P. vivax Genome Variation Project, Wellcome Open Res 2022	23
Cambodia	ERR123849	MalariaGEN P. vivax Genome Variation Project, Wellcome Open Res 2022	23
Cambodia	ERR152408	MalariaGEN P. vivax Genome Variation Project, Wellcome Open Res 2022	23
Cambodia	ERR152410	MalariaGEN P. vivax Genome Variation Project, Wellcome Open Res 2022	23
Cambodia	ERR152413	MalariaGEN P. vivax Genome Variation Project, Wellcome Open Res 2022	23
Cambodia	ERR337538	MalariaGEN P. vivax Genome Variation Project, Wellcome Open Res 2022	23
Cambodia	ERR337542	MalariaGEN P. vivax Genome Variation Project, Wellcome Open Res 2022	23
Cambodia	ERR386533	MalariaGEN P. vivax Genome Variation Project, Wellcome Open Res 2022	23
Cambodia	ERR386534	MalariaGEN P. vivax Genome Variation Project, Wellcome Open Res 2022	23
Cambodia	ERR386535	MalariaGEN P. vivax Genome Variation Project, Wellcome Open Res 2022	23
Cambodia	ERR386536	MalariaGEN P. vivax Genome Variation Project, Wellcome Open Res 2022	23
Cambodia	ERR386537	MalariaGEN P. vivax Genome Variation Project, Wellcome Open Res 2022	23
Cambodia	ERR386538	MalariaGEN P. vivax Genome Variation Project, Wellcome Open Res 2022	23
Cambodia	ERR386539	MalariaGEN P. vivax Genome Variation Project, Wellcome Open Res 2022	23
Cambodia	ERR386541	MalariaGEN P. vivax Genome Variation Project, Wellcome Open Res 2022	23
Cambodia	ERR386542	MalariaGEN P. vivax Genome Variation Project, Wellcome Open Res 2022	23
Cambodia	ERR386543	MalariaGEN P. vivax Genome Variation Project, Wellcome Open Res 2022	23
Cambodia	ERR386546	MalariaGEN P. vivax Genome Variation Project, Wellcome Open Res 2022	23
Cambodia	ERR388742	MalariaGEN P. vivax Genome Variation Project, Wellcome Open Res 2022	23
Cambodia	SRR572648	MalariaGEN P. vivax Genome Variation Project, Wellcome Open Res 2022	23
Indonesia	ERR054085	MalariaGEN P. vivax Genome Variation Project, Wellcome Open Res 2022	23
Indonesia	ERR337624	MalariaGEN P. vivax Genome Variation Project, Wellcome Open Res 2022	23
Indonesia	ERR337627	MalariaGEN P. vivax Genome Variation Project, Wellcome Open Res 2022	23
Indonesia	ERR337630	MalariaGEN P. vivax Genome Variation Project, Wellcome Open Res 2022	23
Malaysia	ERR054088	MalariaGEN P. vivax Genome Variation Project, Wellcome Open Res 2022	23
Malaysia	ERR054089	MalariaGEN P. vivax Genome Variation Project, Wellcome Open Res 2022	23
Malaysia	ERR152414	MalariaGEN P. vivax Genome Variation Project, Wellcome Open Res 2022	23
Malaysia	ERR152415	MalariaGEN P. vivax Genome Variation Project, Wellcome Open Res 2022	23
Malaysia	ERR527337	MalariaGEN P. vivax Genome Variation Project, Wellcome Open Res 2022	23
Malaysia	ERR527363	MalariaGEN P. vivax Genome Variation Project, Wellcome Open Res 2022	23
Thailand	ERR111709	MalariaGEN P. vivax Genome Variation Project, Wellcome Open Res 2022	23
Thailand	ERR111710	MalariaGEN P. vivax Genome Variation Project, Wellcome Open Res 2022	23
Thailand	ERR111711	MalariaGEN P. vivax Genome Variation Project, Wellcome Open Res 2022	23

Thailand	ERR111712	MalariaGEN P. vivax Genome Variation Project, Wellcome Open Res 2022	23
Thailand	ERR111713	MalariaGEN P. vivax Genome Variation Project, Wellcome Open Res 2022	23
Thailand	ERR111714	MalariaGEN P. vivax Genome Variation Project, Wellcome Open Res 2022	23
Thailand	ERR111715	MalariaGEN P. vivax Genome Variation Project, Wellcome Open Res 2022	23
Thailand	ERR111716	MalariaGEN P. vivax Genome Variation Project, Wellcome Open Res 2022	23
Thailand	ERR111717	MalariaGEN P. vivax Genome Variation Project, Wellcome Open Res 2022	23
Thailand	ERR111718	MalariaGEN P. vivax Genome Variation Project, Wellcome Open Res 2022	23
Thailand	ERR111719	MalariaGEN P. vivax Genome Variation Project, Wellcome Open Res 2022	23
Thailand	ERR111720	MalariaGEN P. vivax Genome Variation Project, Wellcome Open Res 2022	23
Thailand	ERR111721	MalariaGEN P. vivax Genome Variation Project, Wellcome Open Res 2022	23
Thailand	ERR111722	MalariaGEN P. vivax Genome Variation Project, Wellcome Open Res 2022	23
Thailand	ERR111723	MalariaGEN P. vivax Genome Variation Project, Wellcome Open Res 2022	23
Thailand	ERR111724	MalariaGEN P. vivax Genome Variation Project, Wellcome Open Res 2022	23
Thailand	ERR111725	MalariaGEN P. vivax Genome Variation Project, Wellcome Open Res 2022	23
Thailand	ERR111726	MalariaGEN P. vivax Genome Variation Project, Wellcome Open Res 2022	23
Thailand	ERR111727	MalariaGEN P. vivax Genome Variation Project, Wellcome Open Res 2022	23
Thailand	ERR111728	MalariaGEN P. vivax Genome Variation Project, Wellcome Open Res 2022	23
Thailand	ERR164695	MalariaGEN P. vivax Genome Variation Project, Wellcome Open Res 2022	23
Thailand	ERR337595	MalariaGEN P. vivax Genome Variation Project, Wellcome Open Res 2022	23
Thailand	ERR337596	MalariaGEN P. vivax Genome Variation Project, Wellcome Open Res 2022	23
Thailand	ERR337597	MalariaGEN P. vivax Genome Variation Project, Wellcome Open Res 2022	23
Thailand	ERR337599	MalariaGEN P. vivax Genome Variation Project, Wellcome Open Res 2022	23
Thailand	ERR337600	MalariaGEN P. vivax Genome Variation Project, Wellcome Open Res 2022	23
Thailand	ERR337601	MalariaGEN P. vivax Genome Variation Project, Wellcome Open Res 2022	23
Thailand	ERR337602	MalariaGEN P. vivax Genome Variation Project, Wellcome Open Res 2022	23
Thailand	ERR337603	MalariaGEN P. vivax Genome Variation Project, Wellcome Open Res 2022	23
Thailand	ERR337604	MalariaGEN P. vivax Genome Variation Project, Wellcome Open Res 2022	23
Thailand	ERR337605	MalariaGEN P. vivax Genome Variation Project, Wellcome Open Res 2022	23
Thailand	ERR337606	MalariaGEN P. vivax Genome Variation Project, Wellcome Open Res 2022	23
Thailand	ERR337607	MalariaGEN P. vivax Genome Variation Project, Wellcome Open Res 2022	23
Thailand	ERR337609	MalariaGEN P. vivax Genome Variation Project, Wellcome Open Res 2022	23
Thailand	ERR337610	MalariaGEN P. vivax Genome Variation Project, Wellcome Open Res 2022	23
Thailand	ERR337611	MalariaGEN P. vivax Genome Variation Project, Wellcome Open Res 2022	23
Thailand	ERR337613	MalariaGEN P. vivax Genome Variation Project, Wellcome Open Res 2022	23
Thailand	ERR337614	MalariaGEN P. vivax Genome Variation Project, Wellcome Open Res 2022	23
Thailand	ERR337616	MalariaGEN P. vivax Genome Variation Project, Wellcome Open Res 2022	23
Thailand	ERR337617	MalariaGEN P. vivax Genome Variation Project, Wellcome Open Res 2022	23
Thailand	ERR337618	MalariaGEN P. vivax Genome Variation Project, Wellcome Open Res 2022	23
Thailand	ERR337619	MalariaGEN P. vivax Genome Variation Project, Wellcome Open Res 2022	23
Thailand	ERR337620	MalariaGEN P. vivax Genome Variation Project, Wellcome Open Res 2022	23
Thailand	ERR337621	MalariaGEN P. vivax Genome Variation Project, Wellcome Open Res 2022	23
Thailand	ERR337622	MalariaGEN P. vivax Genome Variation Project, Wellcome Open Res 2022	23
Thailand	ERR337623	MalariaGEN P. vivax Genome Variation Project, Wellcome Open Res 2022	23
Thailand	ERR337625	MalariaGEN P. vivax Genome Variation Project, Wellcome Open Res 2022	23
Thailand	ERR337626	MalariaGEN P. vivax Genome Variation Project, Wellcome Open Res 2022	23
Thailand	ERR337628	MalariaGEN P. vivax Genome Variation Project, Wellcome Open Res 2022	23
Thailand	ERR404246	MalariaGEN P. vivax Genome Variation Project, Wellcome Open Res 2022	23
Thailand	ERR426015	MalariaGEN P. vivax Genome Variation Project, Wellcome Open Res 2022	23
Thailand	ERR426035	MalariaGEN P. vivax Genome Variation Project, Wellcome Open Res 2022	23
Thailand	ERR527338	MalariaGEN P. vivax Genome Variation Project, Wellcome Open Res 2022	23
Thailand	ERR527339	MalariaGEN P. vivax Genome Variation Project, Wellcome Open Res 2022	23
Thailand	ERR527340	MalariaGEN P. vivax Genome Variation Project, Wellcome Open Res 2022	23
Thailand	ERR527341	MalariaGEN P. vivax Genome Variation Project, Wellcome Open Res 2022	23
Thailand	ERR527342	MalariaGEN P. vivax Genome Variation Project, Wellcome Open Res 2022	23
Thailand	ERR527343	MalariaGEN P. vivax Genome Variation Project, Wellcome Open Res 2022	23
Thailand	ERR527344	MalariaGEN P. vivax Genome Variation Project, Wellcome Open Res 2022	23
Thailand	ERR527345	MalariaGEN P. vivax Genome Variation Project, Wellcome Open Res 2022	23
Thailand	ERR527346	MalariaGEN P. vivax Genome Variation Project, Wellcome Open Res 2022	23

Thailand	ERR527348	MalariaGEN P. vivax Genome Variation Project, Wellcome Open Res 2022	23
Thailand	ERR527350	MalariaGEN P. vivax Genome Variation Project, Wellcome Open Res 2022	23
Thailand	ERR527351	MalariaGEN P. vivax Genome Variation Project, Wellcome Open Res 2022	23
Thailand	ERR527352	MalariaGEN P. vivax Genome Variation Project, Wellcome Open Res 2022	23
Thailand	ERR527353	MalariaGEN P. vivax Genome Variation Project, Wellcome Open Res 2022	23
Thailand	ERR527355	MalariaGEN P. vivax Genome Variation Project, Wellcome Open Res 2022	23
Thailand	ERR527356	MalariaGEN P. vivax Genome Variation Project, Wellcome Open Res 2022	23
Thailand	ERR527358	MalariaGEN P. vivax Genome Variation Project, Wellcome Open Res 2022	23
Thailand	ERR527362	MalariaGEN P. vivax Genome Variation Project, Wellcome Open Res 2022	23
Thailand	ERR527364	MalariaGEN P. vivax Genome Variation Project, Wellcome Open Res 2022	23
Thailand	ERR527365	MalariaGEN P. vivax Genome Variation Project, Wellcome Open Res 2022	23
Thailand	ERR527366	MalariaGEN P. vivax Genome Variation Project, Wellcome Open Res 2022	23
Thailand	ERR527367	MalariaGEN P. vivax Genome Variation Project, Wellcome Open Res 2022	23
Thailand	ERR527368	MalariaGEN P. vivax Genome Variation Project, Wellcome Open Res 2022	23
Thailand	ERR527369	MalariaGEN P. vivax Genome Variation Project, Wellcome Open Res 2022	23
Thailand	ERR527370	MalariaGEN P. vivax Genome Variation Project, Wellcome Open Res 2022	23
Thailand	ERR527371	MalariaGEN P. vivax Genome Variation Project, Wellcome Open Res 2022	23
Thailand	ERR527372	MalariaGEN P. vivax Genome Variation Project, Wellcome Open Res 2022	23
Thailand	ERR527374	MalariaGEN P. vivax Genome Variation Project, Wellcome Open Res 2022	23
Thailand	ERR527375	MalariaGEN P. vivax Genome Variation Project, Wellcome Open Res 2022	23
Thailand	ERR527376	MalariaGEN P. vivax Genome Variation Project, Wellcome Open Res 2022	23
Thailand	ERR527377	MalariaGEN P. vivax Genome Variation Project, Wellcome Open Res 2022	23
Thailand	ERR527379	MalariaGEN P. vivax Genome Variation Project, Wellcome Open Res 2022	23
Thailand	ERR527380	MalariaGEN P. vivax Genome Variation Project, Wellcome Open Res 2022	23
Thailand	ERR527381	MalariaGEN P. vivax Genome Variation Project, Wellcome Open Res 2022	23
Thailand	ERR527382	MalariaGEN P. vivax Genome Variation Project, Wellcome Open Res 2022	23
Thailand	ERR527383	MalariaGEN P. vivax Genome Variation Project, Wellcome Open Res 2022	23
Thailand	ERR527384	MalariaGEN P. vivax Genome Variation Project, Wellcome Open Res 2022	23
Thailand	ERR527385	MalariaGEN P. vivax Genome Variation Project, Wellcome Open Res 2022	23
Vietnam	ERR021980	MalariaGEN P. vivax Genome Variation Project, Wellcome Open Res 2022	23
Vietnam	ERR021981	MalariaGEN P. vivax Genome Variation Project, Wellcome Open Res 2022	23
Vietnam	ERR021982	MalariaGEN P. vivax Genome Variation Project, Wellcome Open Res 2022	23
Vietnam	ERR021983	MalariaGEN P. vivax Genome Variation Project, Wellcome Open Res 2022	23
Vietnam	ERR022866	MalariaGEN P. vivax Genome Variation Project, Wellcome Open Res 2022	23
Vietnam	ERR023161	MalariaGEN P. vivax Genome Variation Project, Wellcome Open Res 2022	23
Vietnam	ERR171580	MalariaGEN P. vivax Genome Variation Project, Wellcome Open Res 2022	23
Vietnam	ERR175494	MalariaGEN P. vivax Genome Variation Project, Wellcome Open Res 2022	23
Vietnam	ERR216527	MalariaGEN P. vivax Genome Variation Project, Wellcome Open Res 2022	23
Vietnam	ERR388796	MalariaGEN P. vivax Genome Variation Project, Wellcome Open Res 2022	23
Papua New Guinea	ERR022864	MalariaGEN P. vivax Genome Variation Project, Wellcome Open Res 2022	23
Papua New Guinea	ERR034096	MalariaGEN P. vivax Genome Variation Project, Wellcome Open Res 2022	23
Papua New Guinea	ERR175552	MalariaGEN P. vivax Genome Variation Project, Wellcome Open Res 2022	23
Papua New Guinea	ERR527453	MalariaGEN P. vivax Genome Variation Project, Wellcome Open Res 2022	23
Papua New Guinea	ERR527467	MalariaGEN P. vivax Genome Variation Project, Wellcome Open Res 2022	23
Papua New Guinea	ERR527468	MalariaGEN P. vivax Genome Variation Project, Wellcome Open Res 2022	23
Papua New Guinea	ERR175555	MalariaGEN P. vivax Genome Variation Project, Wellcome Open Res 2022	23
Papua New Guinea	ERR175557	MalariaGEN P. vivax Genome Variation Project, Wellcome Open Res 2022	23
Ethiopia	ERR2678989	Hupalo et al., Nature Genetics 2016	26
Ethiopia	ERR2678991	Hupalo et al., Nature Genetics 2016	26
Ethiopia	ERR2678992	Hupalo et al., Nature Genetics 2016	26
Ethiopia	ERR2678993	Hupalo et al., Nature Genetics 2016	26
Ethiopia	ERR2678994	Hupalo et al., Nature Genetics 2016	26
Ethiopia	ERR2678996	Hupalo et al., Nature Genetics 2016	26
Ethiopia	ERR2678998	Hupalo et al., Nature Genetics 2016	26
Ethiopia	ERR2678999	Hupalo et al., Nature Genetics 2016	26
Ethiopia	ERR2679000	Hupalo et al., Nature Genetics 2016	26
Ethiopia	ERR2679002	Hupalo et al., Nature Genetics 2016	26
Ethiopia	ERR2679003	Hupalo et al., Nature Genetics 2016	26

Ethiopia	ERR2679004	Hupalo et al., Nature Genetics 2016	26
Ethiopia	ERR2679005	Hupalo et al., Nature Genetics 2016	26
Ethiopia	ERR2679006	Hupalo et al., Nature Genetics 2016	26
Ethiopia	ERR2679007	Hupalo et al., Nature Genetics 2016	26
Ethiopia	ERR2679008	Hupalo et al., Nature Genetics 2016	26
Ethiopia	ERR2679009	Hupalo et al., Nature Genetics 2016	26
Ethiopia	ERR2679010	Hupalo et al., Nature Genetics 2016	26
Ethiopia	ERR2679012	Hupalo et al., Nature Genetics 2016	26
Ethiopia	ERR775189	Hupalo et al., Nature Genetics 2016	26
Ethiopia	ERR775190	Hupalo et al., Nature Genetics 2016	26
Ethiopia	ERR775191	Hupalo et al., Nature Genetics 2016	26
Ethiopia	ERR775192	Hupalo et al., Nature Genetics 2016	26
Ethiopia	ERR925409	Hupalo et al., Nature Genetics 2016	26
Ethiopia	ERR925410	Hupalo et al., Nature Genetics 2016	26
Ethiopia	ERR925411	Hupalo et al., Nature Genetics 2016	26
Ethiopia	ERR925412	Hupalo et al., Nature Genetics 2016	26
Ethiopia	ERR925416	Hupalo et al., Nature Genetics 2016	26
Ethiopia	ERR925417	Hupalo et al., Nature Genetics 2016	26
Ethiopia	ERR925420	Hupalo et al., Nature Genetics 2016	26
Ethiopia	ERR925421	Hupalo et al., Nature Genetics 2016	26
Ethiopia	ERR925424	Hupalo et al., Nature Genetics 2016	26
Ethiopia	ERR925430	Hupalo et al., Nature Genetics 2016	26
Ethiopia	ERR925431	Hupalo et al., Nature Genetics 2016	26
Ethiopia	ERR925434	Hupalo et al., Nature Genetics 2016	26
Ethiopia	ERR925435	Hupalo et al., Nature Genetics 2016	26
Ethiopia	ERR925436	Hupalo et al., Nature Genetics 2016	26
Ethiopia	ERR925437	Hupalo et al., Nature Genetics 2016	26
Ethiopia	ERR925438	Hupalo et al., Nature Genetics 2016	26
Ethiopia	ERR925439	Hupalo et al., Nature Genetics 2016	26
Ethiopia	ERR925440	Hupalo et al., Nature Genetics 2016	26
Ethiopia	ERR925441	Hupalo et al., Nature Genetics 2016	26
Mauritania	SRR332410	Wurtz et al., Malaria Journal 2011	14
Mauritania	SRR332413	Wurtz et al., Malaria Journal 2011	14
Mauritania	SRR340129	Wurtz et al., Malaria Journal 2011	14
Madagascar	SRR570031	Menard et al., PLoS Negl Trop Dis 2013	16
Madagascar	ERR490350	Menard et al., PLoS Negl Trop Dis 2013	16

3.2. Table S2: Distribution of monoclonal (Fws > 0.95) and polyclonal (Fws ≤ 0.95) isolates, by country of origin.

**Country**	**No. of isolates**	**monoclonal isolates**	**polyclonal isolates**
Brazil	5	4	1
Cambodia	29	16	13
Columbia	5	3	2
Comores	8	8	0
Djibouti	2	2	0
Ethiopia	83	57	26
Indonesia	4	1	3
Madagascar	35	21	14
Malaysia	6	6	0
Mauritania	8	8	0
Papua_New_Guinea	8	8	0
Thailand	90	59	31
Vietnam	10	5	5
Total	293	198	95

3.3. Table S3. Mutation points detected in genes associated with drug resistance in *P. vivax* isolates collected in the Comoros, Madagascar, Mauritania, Ethiopia and Djibouti.

Gene	SNPs	Comoros	Madagascar	Mauritania	Ethiopia	Djibouti	Total (%)
	No. of sample	6	24	3	34	1	68
PVP01_0526600 *(bifunctional dihydrofolate reductase-thymidylate synthase, putative)*	WT	5	3	2	34	1	45 (66%)
	C49R		18				18 (26%)
	N130K		3				3 (4%)
	P33L	1					1 (1%)
	A255T			1			1 (1%)
PVP01_1429500 *(hydroxymethyldihydropterin pyrophosphokinase-dihydropteroate synthase, putative)*	WT		22				22 (32%)
	E142G/M205I/G383A				14	1	15 (22%)
	G383A	3	2		10		15 (22%)
	E142G/M205I/A647V				10		10 (15%)
	M205I/G383A	2		2			4 (6%)
	M205I/G383A/**I545T**			1			1 (1%)
	G383A/A647V	1					1 (1%)
PVP01_1010900 *(ABC transporter B family member 1, putative, multidrug resistance protein 1, putative)*	WT	6	23	0	2		31 (46%)
	F976Y				24	1	25 (37%)
	S698G/F976Y			1	6		7 (10%)
	L845F/F976Y			2			2 (2%)
	**F194Y**		1				1 (1%)
	S698G				1		1 (1%)
	T1269S				1		1 (1%)

Novel SNPs found, never described before, are shown in bold

3.4. Table S4. Mutation points detected in invasion-related genes in *P. vivax* isolates collected in the Comoros, Madagascar, Mauritania, Ethiopia, and Djibouti.

Gene	SNPs	Comoros	Madagascar	Mauritania	Ethiopia	Djibouti	Total
	No. of sample	6	24	3	34	1	68
PVP01_0102300 *(erythrocyte binding protein/duffy binding protein 2)*	WT	3	15	2	33	1	54 (79%)
	**I611F**	1	7				8 (12%)
	D268N/E341K	1	1				2 (3%)
	D268N/**F746I**			1			1 (1%)
	**K595N**		1				1 (1%)
	E660K				1		1 (1%)
	**V705L**	1					1 (1%)
PVP01_0623800 *(duffy binding protein)*	WT	5	23	2	34	1	65 (96%)
	**K277T**	1	1				2 (3%)
	**G830V**			1			1 (1%)
PVP01_0800700 *(reticulocyte binding protein 2b)*	WT	5	23	3	34	1	66 (97%)
	**K112I**	1	1				2 (3%)
PVP01_1402400 *(reticulocyte binding protein 2a)*	WT	4	10	3	34	1	52 (76%)
	S186N	1	6				7 (10%)
	L84F		5				5 (7%)
	D461G	1	3				4 (6%)

Novel SNPs found, never described before, are shown in bold

3.5. Table S5. List of validated and suspected *P. vivax* invasion-related genes used in this study.

Ligands	Accession number (*Pv*P01)	Role in invasion	Location	Size
MSP1	PVP01_0728900	confirmed	PvP01_07_v1:1,215,432..1,220,636(+)	5205 bp
GAMA	PVP01_0505600	potential	PvP01_05_v1:253,743..255,818(+)	3415 bp utr
MSP1P	PVP01_0728800	potential	PvP01_07_v1:1,206,976..1,212,549(+)	5574 bp
DBP	PVP01_0623800	confirmed	PvP01_06_v1:982,025..985,813(+)	5979 bp intron utr
EBP/DBP2	PVP01_0102300	potential	PvP01_01_v1:104,127..107,068(-)	3417 bp intron utr
RBP2b	PVP01_0800700	confirmed	PvP01_08_v1:34,100..42,108(+)	10393 bp intron utr
RBP2a	PVP01_1402400	confirmed	PvP01_14_v1:113,324..120,966(+)	8696 bp intron utr
RBSA	PVP01_0004240	potential	Transfer.PvP01_00_11.final:23,781..25,244(+)	1464 bp intron
P12	PVP01_1136400	potential	PvP01_11_v1:1,556,940..1,558,028(+)	1697 bp utr
AMA1	PVP01_0934200	potential	PvP01_09_v1:1,459,296..1,460,984(+)	2784 bp utr
RON	PVP01_0916600	potential	PvP01_09_v1:744,059..746,709(-)	3640 bp intron utr
ETRAMP	PVP01_0532300	potential	PvP01_05_v1:1,361,360..1,362,476(-)	6030 bp intron utr
MSA180	PVP01_0814200	potential	PvP01_08_v1:613,890..618,680(+)	6438 bp utr
RAMA	PVP01_0107500	potential	PvP01_01_v1:375,340..378,546(+)	295 bp intron utr

3.6. Table S6. Primer sequences and PCR conditions used in the study.

Assay	Primer	Sequences (5'-3')	Master mix	Parameters	T°C melt peak
Duffy Genotyping	Duffy	F: AATCCAACCTCAAAACAGGA<br>R: CCCAAATTCCCACAGTGA	5µl DNA template, 200nM primers (Sigma Aldrich), 1X Hot FirePol PCR mix (Solis BioDyne) for total volume 50µl	94°C for 15min<br>94°C for 30s x 40 cycles<br>60°C for 40s x 40 cycles<br>72°C for 90s x 40 cycles<br>72°C for 10min	NA
	GATA-1	F: GAGGCTTGTGCAGGCAGT<br>R: CAAACAGCAGGGGAAATGAG	5µl PCR product diluted (1:10), 200nM primers (Sigma Aldrich), 1X Hot FirePol PCR mix (Solis BioDyne) for total volume 50µl		
	FY	F: CCCTCAATTCCCAGGAGACT<br>R: GCTGAGCCATACCAGACACA			
Screening	screenin g	F: TGGAGTGGATGGTGTTTTAGA<br>R: TTGCACCCCAATARCTCATTT	5µl DNA template, 150nM primers (Sigma Aldrich), 1X Hot FirePol EvaGreen qPCR mix (Solis BioDyne) for total volume 20µl	95°C for 15min<br>95°C for 15s x 45 cycles<br>60°C for 20s x 45 cycles<br>72°C for 20s x 45 cycles<br>95°C for 2min<br>68°C for 2min<br>Increment 0.2°C/0.05s from 68°C to 90°C	76.4 - 78.4°C
Species	All species	F: TGGAGTGGATGGTGTTTTAGA<br>R: ACCCTAAAGGATTTGTGCTACC	5µl DNA template, 250nM primers (Sigma Aldrich), 1X Hot FirePol PCR mix (Solis BioDyne) for total volume 20µl	94°C for 15min<br>94°C for 30s x 20 cycles<br>58°C for 60s x 20 cycles<br>72°C for 60s x 20 cycles<br>72°C for 10min	NA
	Pf	F: ATGGATATCTGGATTGATTTTATTTATGA<br>R: TCCTCCACATATCCAAATTACTGC	5µl PCR products diluted (1:10), 250nM primers, 1X Hot FirePol EvaGreen HRM mix (Solis BioDyne) for total volume 20µl	95°C for 15min<br>95°C for 10s x 40 cycles<br>62°C for 20s x 40 cycles<br>72°C for 25s x 40 cycles<br>95°C for 1min<br>40°C for 1min	78.6 - 79.6°C
	Pv/Pk	F: TGCTACAGGTGCATCTCTTGTATTC<br>R: ATTTGTCCCCAAGGTAAAACG			74.8-75.8°C
	Pm	F: ACAGGTGCATCACTTGTATTTTTTC<br>R: TGCTGGAATTGAAGATAATAAATTAGTAATAACT			75.4 - 76.4°C
	Po	F: GTTATATGGTTATGTGGAGGATATACTGTT<br>R: CGAATGGAAGAATAAAATGTAGTACG			73.2 -74.2°C

sWGA	Set 1	pvS1: CGTTG*C*G<br>pvS2: TTTTTTC*G*C<br>pvS3: TCGTG*C*G<br>pvS4 : CGTTTTTT*T*T<br>pvS5 : TTTTTTTC*G*T<br>pvS6 : CCGTT*C*G<br>pvS7 : CGTTTC*G*T<br>pvS8 : CGTTTC*G*C<br>pvS9 : CGTTTT*C*G<br>pvS10 : TCGTTC*G*T	12.5µl DNA template, 3.5µM primers (Sigma Aldrich), 4mM dNTPs (Biotech biobasic), 30U phi29 DNA polymerase (New England Biolabs), 1X phi29 buffer (New England Biolabs), 1% BSA (Invitrogen) for total volume 50µl<br><br>The * indicate phosphorothioate bonds that are necessary to prevent degradation by phi29 DNA polymerase	35°C for 10min<br>34°C for 10min<br>33°C for 10min<br>32°C for 10min<br>31°C for 10min<br>30°C for 10min<br>30°C for 16h<br>65°C for 10min	NA
	Set 1920	pvS1: AACGAAGC*G*A<br>pvS2: ACGAAGCG*A*A<br>pvS3: ACGACGA*A*G<br>pvS4: ACGCGCA*A*C<br>pvS5: CAACGCG*G*T<br>pvS6: GACGAAA*C*G<br>pvS7: GCGAAAAA*G*G<br>pvS8: GCGAAGC*G*A<br>pvS9: GCGGAAC*G*A<br>pvS10: GCGTCGA*A*G<br>pvS11: GGTTAGCG*G*C<br>pvS12: AACGAAT*C*G			

4. References

1. Auburn S, *et al.* Characterization of within-host Plasmodium falciparum diversity using next-generation sequence data. *PLoS One* **7**, e32891 (2012).
2. Van der Auwera & O'Connor. *Genomics in the Cloud.* https://www.oreilly.com/library/view/genomics-in-the/9781491975183/ (accessed 2023-05-01).
3. Li H. Aligning Sequence Reads, Clone Sequences and Assembly Contigs with BWA-MEM. arXiv 2013. https://doi.org/10.48550/arXiv.1303.3997.
4. Raj A., *et al.* FastSTRUCTURE: Variational Inference of Population Structure in Large SNP Data Sets. *Genetics* 2014, *197* (2), 573–589. htps://doi.org/10.1534/genetics.114.164350.
